# Improved quality of life after surgery for pelvic organ prolapse in Nepalese women

**DOI:** 10.1186/1472-6874-13-22

**Published:** 2013-05-09

**Authors:** Rolina Dhital, Keiko Otsuka, Krishna C Poudel, Junko Yasuoka, Ganesh Dangal, Masamine Jimba

**Affiliations:** 1 Department of Community and Global Health, Graduate School of Medicine, The University of Tokyo, 7-3-1, Hongo, Bunkyo-ku, Tokyo, 113-0033, Japan; 2 Department of Public Health, School of Public Health and Health Sciences, University of Massachusetts Amherst, 316 Arnold House, 715 North Pleasant St, Amherst, MA, 01003-9304, USA; 3 Department of Obstetrics and Gynecology, Kathmandu Model Hospital, P.O. Box 6064, Exhibition Road, Kathmandu, Nepal

**Keywords:** Nepal, Pelvic organ prolapse, Quality of life, Surgery, Vaginal hysterectomy

## Abstract

**Background:**

Pelvic organ prolapse (POP) is a common gynecological condition that can affect quality of life (QOL) in women. In Nepal, the prevalence of POP is high, but many affected women are still deprived of treatment. Vaginal hysterectomy with pelvic floor repair is one of the common treatment options for advanced POP. However, QOL outcomes after surgery have not been reported in low-income countries. Thus, we aimed to examine changes in QOL among Nepalese women with POP after such surgery.

**Methods:**

This longitudinal study was conducted in the selected central and peripheral hospitals in Nepal where vaginal hysterectomy was being performed free of cost for POP. A baseline study first measured the QOL domains (physical, psychological, social relationships and environment) among 252 women with advanced POP. Follow-up data was then collected at six weeks and three months after surgery. Among the 177 women that were available at six weeks post-surgery, 166 participated in the follow-up study at three months post-surgery. To evaluate QOL at baseline, 142 women with no history of POP were included as a comparison group.

**Results:**

The mean scores across QOL domains improved from baseline to 3 months after surgery. The baseline score for the physical domain increased from 11.2 to 12.8 at six weeks and 13.5 at three months post-surgery (*p* < 0.001); the psychological domain score increased from 11.6 to 13.1 at six weeks and 13.8 at three months post-surgery (*p* < 0.001); the social relationships domain score increased from 13.6 to 14.4 at six weeks and 15.0 at three months post-surgery (*p* < 0.001); and the environmental domain score increased from 12.9 to 13.9 at six weeks and 14.0 at three months post-surgery (*p* < 0.001).

**Conclusion:**

QOL progressively improved among women undergoing surgery for POP. Such surgical services need to be scaled up for treatment of advanced POP in low-income countries.

## Background

Pelvic organ prolapse (POP) is a common non-life-threatening gynecological condition. The risk factors for POP include pregnancy, childbirth, weakness of the pelvic floor, aging and menopause [1-3]. In developed countries, the prevalence of POP is high among postmenopausal women [2,4,5], whereas in developing countries, the condition is also common in women of reproductive age [6,7]. The prevalence of POP at the global level is 2%–20% in women under the age of 45 years [8].

In Nepal, the prevalence of POP is high. It exceeds 10% in women of reproductive age and is 24% among women between 45 and 49 years of age [9]. Among women diagnosed with POP, 69.1% have first-degree prolapse while the remaining 30.9% suffer from second- and third-degree prolapse [10].

POP can affect many aspects of quality of life (QOL) in women [2,11,12]. It can result in physical, psychological, social and sexual lifestyle limitations [12]. In low-income countries like Nepal, discrimination against women with POP by family and society at large is common [6,13]. This may further worsen their QOL. Affected women often tend to isolate themselves from social activities due to shame and fear of abandonment.

Although POP is treatable, many women do not receive timely treatment in many low-income countries. This could be due to lack of awareness about treatment options, lack of control over when to seek medical treatment, and inability to pay for medical expenses or transportation costs to access health facilities [6]. Furthermore, the human resources and other treatment facilities are limited in many remote health centers. In Nepal, only approximately 25% of women with POP receive treatment because of these barriers to health care [14].

In general, treatment of POP varies depending on the extent of the pelvic organs’ descent, the reported symptoms, the woman’s general health, and the surgeon’s preference and competences. Available treatment options include conservative, mechanical or surgical interventions. Surgical treatment is generally considered for women with symptomatic prolapse, those medically fit for surgery, and those willing to undergo surgery [15]. Vaginal hysterectomy is one of several surgical procedures to treat advanced POP [16]. It is also one of the most common reasons for hysterectomy in women over the age of 55 years [12].

The government of Nepal created a fund to provide free surgery to women with POP in 2008 [17]. In 2010 and 2011, the Ministry of Health’s Family Health Division in collaboration with the United Nations Population Fund (UNFPA) used the fund to treat 13,000 women with advanced POP at an operative cost of 150 US dollars per case [17]. Mobile surgery camps for POP represent one approach for providing such services to rural, remote and underserved populations in Nepal.

In the context of Nepal, surgery camp refers to the surgeries conducted by an expert team within a specified period time, in a specified place. In order to have a wide coverage, these surgery camps are conducted both in central and peripheral health facilities. The stakeholders and the funded hospitals that host the surgeries generally decide the date and location of camps to be organized. The surgeries are provided free of charge and each patient, along with one attendant, is granted travel and food expenses. Vaginal hysterectomy with pelvic floor repair is generally the treatment of choice for women with advanced POP in these surgery camps.

Studies on QOL outcomes after surgery for POP are still limited, particularly in the developing country context. A few studies have been conducted to measure QOL before and after a hysterectomy for advanced POP in developed countries [4,11,18]. In low-income countries, however, no such post-surgical treatment QOL study has been reported. Preliminary studies from Nepal suggest that POP may negatively influence women’s QOL [3,13,19]. However, quantitative evidence is still lacking. Thus, we conducted this study to examine the change in QOL among Nepalese women with advanced POP before and after surgery.

## Methods

### Study settings

We conducted this longitudinal study in three surgery camps in Kathmandu, Dolakha and Dadeldhura districts of Nepal. Camps included in our study were organized by the Public Health Concern Trust, Nepal (phect-NEPAL) in collaboration with the government of Nepal, with financial support provided by the Ministry of Health and Population. We included all surgeries that took place at Kathmandu Model Hospital in Kathmandu, Gaurishankar General Hospital in Dolakha, and Team Hospital in Dadeldhura. The surgical team from phect-NEPAL provided the services at all selected sites.

From each study site, we included all women with third- or fourth-degree POP [20], each of whom underwent vaginal hysterectomy with pelvic floor repair following a standard pre-anesthetic checkup. All surgeries were performed under spinal anesthesia. No major intra- or post-operative complications were recorded in the three selected surgery camps, and intra- and post-operative management procedures were standard across the sites. All patients were discharged from the health facilities on the fifth post-operative day with prescribed analgesics, antibiotics and other medications as needed.

The health workers from the surgery camps provided counseling regarding post-operative care. All patients who underwent surgeries between February 2011 and June 2011 were included in the study. In addition to surgery, the camp facilities also provided screening for POP and reproductive health education to both women with and those without POP.

### Participants

We conducted baseline interviews with participating women at the time of diagnosis, at which time they were also scheduled for surgery in the camps. Follow-up interviews were conducted at six weeks and three months post-surgery.

To evaluate QOL at baseline, we also included women with no history of POP as a comparison group. We included this comparison group in order to assess how QOL among women with POP differs from that of women without POP. Women with even a slight degree of POP were excluded from the comparison group to reduce potential bias, as even mild POP could have possibly influenced their QOL. These women were recruited from the same surgery camp settings when they came for general check-ups, visited other patients, or participated in reproductive health education programs. Women currently under medication for diagnosed psychiatric illnesses or comorbidities were excluded from both groups.

**Figure 1:**
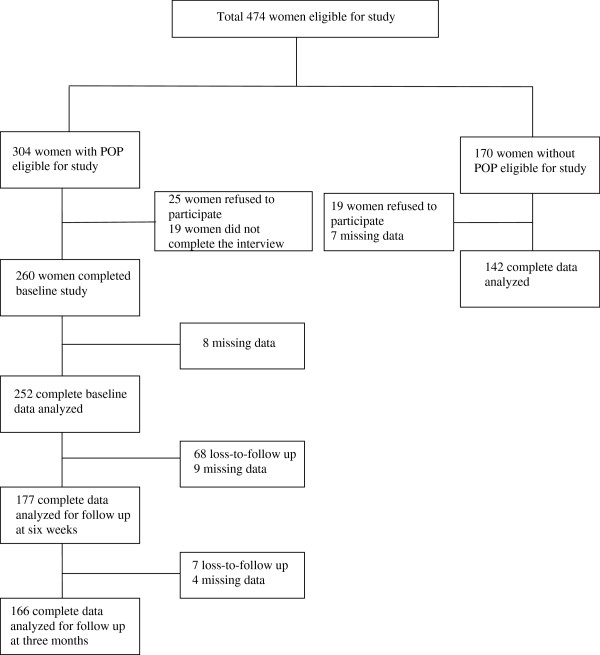
Study participants flow.

In total, 304 women with POP provided informed consent to participate in the study, of whom 260 completed the interview at baseline. However, due to missing information, we included complete data from 252 women in the baseline analysis. Follow-up was conducted by a telephone-based interview after surgery. Out of the 177 women that completed the survey at six weeks, 166 completed the survey-based interview at three months (Figure 1). As a result, the completion rate from baseline to the three-month follow-up was 64%. With regard to the comparison group, 170 women provided informed consent to participate, of whom 142 completed the interview.

### Measures

We used the WHOQOL-BREF, a 26-item abbreviated version derived from the WHOQOL-100, to measure participants’ QOL [21]. This tool generates scores in four domains of QOL – physical, psychological, social and environmental health. The item scores range from 1 to 5, with a higher score indicating better QOL. The WHOQOL-BREF has been translated into the Nepali language, and the translated version showed good internal reliability (Cronbach’s alpha = 0.85) in a study of QOL among people living with HIV/AIDS [22].

We used Beck’s Depression Inventory I-A to assess depression. The 21 items of this instrument are coded on a 4-point Likert scale, with the total score ranging from 0 to 63. Higher total scores indicate more severe depressive symptoms. The Nepali version of the scale has been validated for use in Nepal [23].

Measured demographic characteristics included age, ethnicity, literacy, marital status, parity and socioeconomic status. We assessed socioeconomic status by a weighted wealth index [24]. The index assessed housing characteristics including floor, wall and roof material; ownership of agricultural land, livestock, herds and other farm animals; and household assets including televisions, radios, clocks, fans, and mobile and non-mobile phones. These items were dichotomized and factor analysis was conducted via principal component analysis to reduce the items from 40 to 24 (loaded as factor 1). Factor loadings were used as item weights and summed to generate the household wealth index. The total wealth index score was then divided into four categories: poorest, poorer, middle income, and richer.

We designated ethnicity on the basis of the participants’ last names, which typically indicate ethnicity and caste in Nepal. We then classified ethnicity into three broader groups: Brahmin/Chhetri, Janajati, and Dalit [25].

### Statistical analysis

We analyzed the data using both descriptive and multivariate analysis. For the descriptive analysis we conducted independent sample t-tests and chi-square tests to compare socio-demographic characteristics, depression and QOL between women with and those without POP. We then conducted multiple linear regression analysis to examine the factors associated with QOL in all participants.

We used a generalized estimating equation (GEE) model to examine changes in QOL among women with POP from baseline to three months after surgery. In this model, we controlled for time and other socio-demographic characteristics.

We used PASW Statistics 18.0 (SPSS Inc., Chicago, Illinois, USA) for all statistical analyses. A *p* value of less than 0.05 was used as the significance level for all analytical procedures.

### Ethical considerations

The Research Ethics Committees of the Graduate School of Medicine at the University of Tokyo, the Nepal Health Research Council and phect-NEPAL reviewed and approved the study protocol. We informed all participants of the study objectives and recruited only those who gave written consent voluntarily to participate in both baseline and follow-up surveys. Confidentiality was maintained for all participants, with all interviews conducted in a location of secured privacy.

## Results

Table 1 shows the characteristics of women with and without POP at baseline. The mean age was higher in women with POP than in those without (52.0 and 40.6 years, respectively; *p* < 0.001). Illiteracy was more common among women with POP (N = 226, 89.7%) than among those without (N = 45, 31.7%; *p* < 0.001). Women with POP also had higher parity (mean = 5.3, SD 2.17) as compared to women without POP (mean = 2.7, SD 1.71; *p* < 0.001). Women with POP also had higher scores for depression (mean = 23.7, SD 8.7) than those without (mean = 8.6, SD 8.6, *p* < 0.001).

**Table 1:** Baseline characteristics of women participants in pelvic organ prolapse surgery camp

	**Women with pelvic organ prolapse (N = 252)**		**Women without pelvic organ prolapse (N = 142)**		
**Characteristics**	**n (%)**	**Mean (SD)**	**n (%)**	**Mean (SD)**	**p-value**
**Age**		52.0 (11.2)		40.6 (10.1)	**<0.001**
**Socioeconomic status**					
Poor	68 (27.1)		32 (22.5)		0.571
Poorer	114 (45.5)		62 (43.7)		
Medium	36 (14.3)		25 (17.6)		
Richer	33 (13.1)		23 (16.2)		
**Marital status**					
With husband	190 (75.4)		114 (80.3)		**0.010**
Separated	14 (5.6)		15 (10.6)		
Widow	48 (19.0)		13 (9.1)		
**Parity**		5.3 (2.17)		2.7 (1.71)	**<0.001**
**Ethnicity**					
Brahmin/Chhetri	134 (53.2)		76 (53.5)		0.065
Janajati	91 (36.1)		60 (42.3)		
Dalit	27 (10.7)		6 (4.2)		
**Literacy**					
Illiterate	226 (89.7)		45(31.7)		**<0.001**
Literate	26 (10.3)		97(68.3)		
**Total depression Score**		23.7 (8.7)		8.6 (8.6)	**<0.001**
**Quality Of Life domains**					
Physical		11.0 (2.5)		14.4 (3.5)	**<0.001**
Psychological		11.4 (2.3)		13.0 (3.2)	**<0.001**
Social relationships		13.5 (3.7)		14.4 (4.2)	**0.024**
Environment		12.0 (2.4)		13.0 (3.6)	**0.002**
Overall health and quality of life		8.6 (3.4)		13.8 (4.2)	**<0.001**

Table 2 shows the adjusted factors associated with all domains of QOL (including POP status) at baseline. The adjusted factors included POP status, age, marital status, parity, ethnicity, wealth index, literacy and depression. The psychological domain score for women with POP was likely to be lower than that for women without (standardized beta co-efficient [*β*] = −0.147, *p =* 0.028). Similarly, scores for the social relationships (*β* = −0.166, *p* = 0.019) and environmental domains (*β* = −0.207, *p* = 0.002) were also lower in women with POP. No significant difference was observed in scores for the physical domain between the two groups. Age, meanwhile, was significantly associated only with the physical domain (*β* = −0.199, *p* < 0.001), while parity showed no significant associations with any domains of QOL. Other factors significantly associated with all domains of QOL in women with and without POP included marital status, literacy, and depression (Table 2).

**Table 2:** **Factors associated with baseline quality of life**^**a **^**in women with and without pelvic organ prolapse**

	**Physical**		**Psychological**		**Social relationships**		**Environment**	
	**β**	***P***	**β**	***P***	**β**	***P***	**β**	***P***
POP^b^ status								
Without POP^c^								
With POP	0.040	0.488	−0.147	0.028	−0.166	0.019	−0.207	0.002
Age	−0.199	<0.001	−0.066	0.245	0.031	0.603	0.040	0.486
Marital status								
Married^c^								
Separated	−0.109	0.005	−0.159	0.001	−0.229	<0.001	−0.142	0.002
Widow	−0.068	0.098	−0.029	0.547	−0.093	0.068	−0.064	0.189
Parity	0.025	0.587	−0.003	0.954	−0.036	0.524	0.079	0.148
Ethnicity								
Brahmin/Chhetri^c^								
Janajati	−0.012	0.755	0.011	0.804	−0.014	0.773	−0.084	0.071
Dalit	−0.036	0.386	−0.048	0.317	−0.082	0.109	−0.143	0.004
Wealth index	0.030	0.440	−0.005	0.909	−0.038	0.426	0.043	0.349
Literacy	0.210	<0.001	0.211	0.001	0.159	0.014	−0.325	<0.001
Depression	−0.392	<0.001	−0.409	<0.001	−0.317	<0.001	−0.331	<0.001

^a^ Linear regression model.

^b^ Pelvic organ prolapse.

^c^ Reference group.

β Standardized beta coefficient.

*P p*-value.

Table 3 shows the baseline characteristics and QOL of women for whom follow-up data was collected and of those lost to follow-up. There were no significant differences in the mean QOL scores between these two groups. A significant difference was, however, noted in socio-economic status. A higher proportion of women who were lost to follow-up were richer as compared to the ones who were available for follow-up (*p* = 0.004). A significant difference was also noted in depression scores between the two groups (mean 22.5, SD 8.9 VS. 26.1, SD 7.5), with the women lost to follow-up showing higher levels of depression (*p* = 0.006).

**Table 3:** Baseline characteristics of women with pelvic organ prolapse with and without follow-up

	**Women with follow-up (N = 153)**		**Women lost to follow-up (N = 99)**		
**Characteristics**	**n (%)**	**Mean(SD)**	**n (%)**	**Mean (SD)**	**p-value**
**Age**		51.1(11.3)		52.6(11.2)	0.287
**Socioeconomic status**					
Poorest	44(28.6)		24(24.2)		**0.004**
Poorer	79(51.3)		35(35.4)		
Medium	18(11.7)		20(20.2)		
Richer	13(8.4)		23(20.2)		
**Marital status**					
With husband	119(76.8)		74(74.7)		0.217
Separated	11(7.1)		3(3.1)		
Widow	25(9.8)		22(22.2)		
**Parity**		5.3(2.6)		5.3(3.0)	0.985
**Ethnicity**					0.586
Brahmin/Chhetri	85(54.8)		52(52.5)	52.5	
Janajati	56(62.2)		34(34.3)	34.3	
Dalit	14(9.0)		13(13.2)	13.2	
**Literacy**					0.870
Illiterate	140(90.9)		90(90.3)		
Literate	9(9.1)		15(9.7)		
**Depression**		22.5(8.9)		26.1 (7.5)	**0.006**
**Family Support**		20.7(6.9)		19.5(5.1)	0.128
**QOL Domains**					
Physical		11.1(2.6)		10.7(2.2)	0.212
Psychological		11.6(2.5)		11.1(2.0)	0.119
Social relationships		13.8(2.7)		13.0(5.0)	0.620
Environment		12.0(2.4)		12.0(2.4)	0.784

Table 4 shows the changes in mean baseline scores for all QOL domains three months after surgery, as calculated by the GEE models. After adjusting for age, marital status, literacy, ethnicity, and economic status, the magnitude of the predicted changes in the different domains of QOL was statistically significant. For the physical domain, the baseline mean score of 11.2 increased to 12.8 at six weeks and 13.5 at three months after surgery (*p* < 0.001). For the psychological domain, the mean baseline score of 11.6 increased to 13.1 at six weeks and 13.8 at three months after surgery (*p* < 0.001). For the social relationships domain, the mean baseline score of 13.6 increased to 14.4 at six weeks and 15.0 at three months after surgery (*p* < 0.001). For the environmental domain, the mean baseline score of 12.9 increased to 13.9 at six weeks and 14.0 at three months after surgery (*p* < 0.001).

**Table 4:** **Changes in mean quality of life scores**^**a **^**from baseline to three months after surgery**

**Domains**	**Baseline**^**b**^	**six weeks**	**three months**
	**Mean (95% confidence interval)**	**Mean (95% confidence interval)**	**Mean (95% confidence interval)**
**Physical**	11.2 (10.8–11.7)	12.8 (12.3–13.2)**	13.5 (13.1–13.9)**
**Psychological**	11.6 (11.1–12.2)	13.1 (12.7–13.8)**	13.8 (13.2–14.3)**
**Social relationships**	13.6 (13.0 –14.3)	14.4 (14.0–15.4)*	15.0 (14.4–15.7)**
**Environment**	12.9 (12.3 –13.4)	13.9 (13.4–14.6)**	14.0 (13.4–14.6)**
**Overall health and quality of life**	8.8 (8.18–9.52)	13.8 (13.2–14.3)**	13.9 (13.2–14.6)**

^a^ Generalized Estimating equation model, adjusted for age, marital status, ethnicity, literacy, and economic status.

^b^ Baseline scores as reference.

*p < 0.05, **p < 0.001.

The depression score decreased significantly from baseline (23.1) to six weeks (8.6) and three months (3.2) after surgery (*p* < 0.001) in the GEE model. We controlled for time, age, ethnicity and economic status for this model.

## Discussion

This study shows that QOL among women with advanced POP may progressively improve after surgery. The improved QOL scores observed in participants encompassed physical, psychological, social relationships and environment domains. Before the surgery, such women with POP had poorer QOL as compared to women without the condition.

At baseline, women with POP scored lower in the social, and environmental QOL domains than did women without prolapse. In Nepal, women with POP often live in shame because of traditional beliefs regarding their genital issues [6]. When the prolapse is advanced, it is often associated with foul-smelling vaginal discharge that makes it difficult for them to hide the condition. Hence, they hesitate to socialize for fear of abandonment by their family and society at large. These women also frequently experience discrimination from their husbands and mothers-in-law [10,13].

Furthermore, women with prolapse showed lower scores in the psychological domain of QOL. They also had higher depressive symptoms as compared to women without prolapse. In Nepal, the majority of women require permission from a family member such as the husband or the mother-in-law to seek health care [13]. However, women’s illnesses still rank low among family priorities, particularly when the condition is not life threatening [26,27]. Such obstacles and resulting delays in treatment may detrimentally affect their mental health. All these factors may help to explain the women’s scores in psychological, social and environmental aspects of QOL.

Baseline physical domain scores for QOL showed no significant difference between women with and those without POP in this study. This finding differs from the results of a study from the U.S. that used the short-form 36-item scale (SF-36) and yielded lower scores for physical QOL [11]. This difference may be attributed to the use of different QOL measures in these two studies. The WHOQOL-BREF was used in this study because it had been used in a prior study and was shown to be suitable in the specific context of Nepal [22]. This difference may also be attributed to differences in lifestyle between the U.S. and Nepal.

This study also showed various factors associated with QOL for women both with and without POP. Those who were illiterate, were separated from their husbands, and had higher depression scores were more likely to have poorer QOL. In general, illiteracy, marital status and depression have been associated with poor QOL in many health conditions [28-30]. On the other hand, parity, which is a major risk factor for POP [3], was not associated with QOL in our study. This could be because baseline QOL was evaluated only for the past one-year period. Even though parity could have been an important factor leading to POP, it may not have been the direct reason for relatively recent experience of poor QOL.

Following surgery among women with POP, we found that all domains of QOL progressively improved until three months had passed. Depressive symptoms also improved remarkably after surgery. Improved mental health status post-operatively could have positively affected post-operative QOL. Another study from the U.S. similarly found better QOL after surgery because of improved depressive symptoms [18]. In low-income countries, camp-based free surgical services for POP can provide accessible and timely treatment to rural, remote and underserved populations. This in turn could have a positive influence on formerly impaired QOL among those affected.

Recently in Nepal, surgery camps for POP have been criticized for their hectic schedules and the lack of firm evidence for positive outcomes [31]. Many critics have accused the camps of effecting no change or causing poor outcomes as a result of the surgery performed there. These criticisms have arisen largely due to the lack of proper post-operative monitoring [31,32]. The implementation of a prospective follow-up at six weeks and three months post-surgery in this study provides a longitudinal perspective on post-operative QOL in women who have undergone camp-based prolapse surgery.

The findings of this study should be considered in light of several limitations. First, no comparison group was included in the follow-up of women who underwent surgery. However, post-operative QOL among women with POP, who had undergone hysterectomies, would not have been comparable to the available baseline QOL data for women without POP. Since QOL among women post-surgery may not be comparable to women with intact uteri, irrespective of their prolapse status, we did not set a comparison group to assess the changes in QOL over time between the two groups. Thus, we cannot completely rule out the possible effect of changes occurring over time.

Second, a high number of patients were lost to follow-up. Our findings indicated gradually improved post-operative QOL after follow-up. It cannot, therefore, be generalized to those who were lost to follow-up. Women who were lost to follow-up and those who remained in the study had similar but low QOL at the baseline. All participants underwent similar surgical treatment with no post-operative complications. However, we cannot pinpoint the definite factors responsible for loss to follow-up. Thus, the possibility of bias due to loss to follow-up cannot be completely ruled out.

Third, there was a significant age difference between women with POP and those without POP in our study. Matching for age was not possible in our study because most of the available elderly women had some form of prolapse, even though it had not reached the advanced stage. Instead, we have thus treated age as a confounding variable and controlled for it in the linear regression model.

Despite these limitations, this is one of the first studies to report on QOL before and after surgery for POP performed in a camp setting in a low-income country. In Nepal, women may resume their regular physical activities as early as six weeks after surgery. This is because women in rural areas are expected to perform heavy physical work irrespective of their health condition [26]. Despite the continued hardships, improved QOL through three months post-surgery supports the idea that such surgical services should be continued.

Although a three-month follow-up may be a short duration for measuring QOL changes, it nonetheless provides important information regarding this intervention. The results through three months also indicate the importance of sustaining women’s improved QOL in the long run. The treatment addressed in this study involves surgical removal of the uterus for advanced POP to improve affected women’s QOL. However, we did not confirm that QOL was fully restored. Further efforts to encourage lifestyle changes after surgery may be needed to prevent incidence of post-hysterectomy vault prolapse in the long term. Additional longitudinal studies are also warranted to explore the clinical outcomes.

## Conclusions

In conclusion, QOL among women with POP improved progressively at three months after vaginal hysterectomy with pelvic floor repair. In low-income countries such as Nepal, free surgical services targeting disadvantaged women need to be scaled up to improve QOL among women suffering from POP.

## Competing interests

The authors declare that they have no competing interests.

## Authors’ contributions

RD conceived the research questions, designed the study, conducted the fieldwork, analyzed the data, and prepared the manuscript draft. KO was involved in research proposal preparation, planning and analysis of data, and revision of the manuscript for publication. KCP was involved in research proposal preparation, data analysis, and revision of the manuscript. JY was involved in data analysis and manuscript revision. GD was involved in proposal development, logistic preparations for fieldwork, data collection supervision, and manuscript revision. MJ was involved in revision of the research proposal, data analysis, and revision of the manuscript. All authors read and approved the final manuscript draft.

## Pre-publication history

The pre-publication history for this paper can be accessed here:

http://www.biomedcentral.com/1472-6874/13/22/prepub
